# A peptide antagonist of Prep1-p160 interaction improves ceramide-induced insulin resistance in skeletal muscle cells

**DOI:** 10.18632/oncotarget.18286

**Published:** 2017-05-30

**Authors:** Ilaria Cimmino, Virginia Lorenzo, Francesca Fiory, Nunzianna Doti, Serena Ricci, Serena Cabaro, Antonietta Liotti, Luigi Vitagliano, Michele Longo, Claudia Miele, Pietro Formisano, Francesco Beguinot, Menotti Ruvo, Francesco Oriente

**Affiliations:** ^1^ Department of Translational Medicine, Federico II University of Naples and URT “Genomic of Diabetes” of Institute of Experimental Endocrinology and Oncology, National Council of Research (CNR), Naples, Italy; ^2^ Institute of Biostructure and Bioimaging, National Research Council and Interuniversity Research Centre on Bioactive Peptides, Naples, Italy

**Keywords:** ceramide, Prep1, p160, Prep1(54-72) peptide, insulin signalling

## Abstract

Prep1 is a homeodomain transcription factor belonging to the TALE protein family. Its overexpression affects glucose metabolism in several tissues. In particular, in skeletal muscle tissue the interaction of Prep1 with its cofactor p160 impairs GLUT4 expression and glucose uptake.

In this study, we show that ceramides (C2cer), a class of lipids antagonizing insulin signalling, increase the levels of Prep1 and p160 in a dose and time-dependent fashion in L6 cells and induce their association by 80%. We find that C2cer exposure inhibits insulin receptor, IRS1 and Akt phosphorylation and reduces insulin-stimulated glycogen content and glucose uptake by 1.3- and 2.1-fold, respectively. The synthetic Prep1(54-72) peptide, mimicking the Prep1 region involved in the interaction with p160, reduces *in vitro* Prep1-p160 binding in a dose-dependent way (IC_50_ = 0.20μM). In C2cer-treated L6 cells, 10μM Prep1(54-72) restores insulin signalling impaired by ceramide treatment. Prep1 overexpressing L6 cells display similar metabolic alterations observed in ceramide-treated L6 cells and the presence of Prep1(54-72) mitigates these events. All these findings suggest that disruption of the Prep1/p160 molecular interaction enhances insulin sensitivity impaired by ceramides in skeletal muscle cells and indicate this complex as an important target for type 2 diabetes.

## INTRODUCTION

Insulin resistance (Ir) is a key etiological factor for type 2 diabetes mellitus (T2DM) which has reached epidemic proportions and is determined by the interaction of genetic and environmental factors, including obesity [1]. Several mechanisms have been proposed to explain the relationship between increased adiposity and Ir. An intriguing hypothesis is that non-adipose tissues, such as muscle and liver, accumulate fatty acids when adipose stores are saturated, inducing lipotoxicity that favors the onset of insulin resistance. Amongst the myriad bioactive lipids that can accumulate in non-adipose tissues, palmitate-derived ceramides have been shown to increase in obese subjects, to induce insulin resistance and to contribute to the development of nonalcoholic fatty liver disease and progression to nonalcoholic steatohepatitis [2–9]. Reducing the levels of these lipids by thiazolidinediones (TZDs) delays or prevents metabolic disease onset [10, 11]. Although TZDs are usually well tolerated, their use may be associated with several adverse effects including bone loss, cardiovascular heart failure and bladder cancer [12–14]. Thus, new pharmacological and genetic therapeutic strategies that inhibit ceramide action by unconventional mechanisms might be perceived as convenient complements or alternatives for treating insulin resistance and T2DM.

Prep (Pbx regulating protein) 1 is a transcription factor belonging to the MEINOX subfamily of the TALE protein family. Prep1 regulates metabolic response in several organs and tissues [15]. Near the C-terminus, Prep1 has a homeodomain region characterized by a 3 aminoacid loop extension (TALE) necessary for DNA binding. In the N-terminus there are sequences similar to MEIS named HR (homology region) 1 and 2, which are essential for the association with other transcription factors such as Pbx1 and p160. Pbx1 is a predominant partner of Prep1 in various tissues and formation of Prep1/Pbx1 dimeric complex increases the stability and the activity of Pbx1 [16, 17]. Indeed, Prep1 hypomorphic heterozygous (*Prep1^i/+^*) mice, expressing low levels of protein, show a strong reduction of pancreatic Pbx1 expression, islet hypoplasia and insulin secretion [18, 19]. However, despite the lowered insulin levels, *Prep1^i/+^* mice are protected from streptozotocin-induced diabetes and feature reduced lipotoxicity and diet-induced steatohepatitis [20]. These effects are due to a better peripheral insulin sensitivity. More detailed studies have indicated that both Pbx1 and p160 compete for the same Prep1 binding site, however Pbx1 levels are higher in liver, while p160 is preponderant in skeletal muscle, suggesting that different mechanisms operate in hepatocytes and in muscle cells. Indeed, in liver, Prep1/Pbx1 restrains insulin action by activating the transcription of the SHP1 tyrosine phosphatase gene and inhibiting insulin receptor and IRS signaling [19]. Preliminary data also suggest that the Prep1-Pbx1 interaction is important for adipocyte differentiation. In muscles, Prep1 binds and stabilizes p160, repressing the PGC-1α-GLUT4-mediated glucose uptake [18].

In this way, disrupting the interaction between Prep1 and its cofactors by selective inhibitors may represent a novel strategy to improve insulin sensitivity in target cells.

In the current work, we show that, in L6 skeletal muscle cells, ceramides increase the levels of Prep1 and of its cofactor p160 and promote their association. Incubation of cells with the synthetic Prep1(54-72) peptide, comprising the 63-70 (63LFPLLALL70) region of Prep1 HR1 domain, inhibits the binding with p160 and restores the metabolic equilibrium altered by ceramides.

## RESULTS

### Ceramide induces Prep1 and p160 expression and association

**Figure 1:**
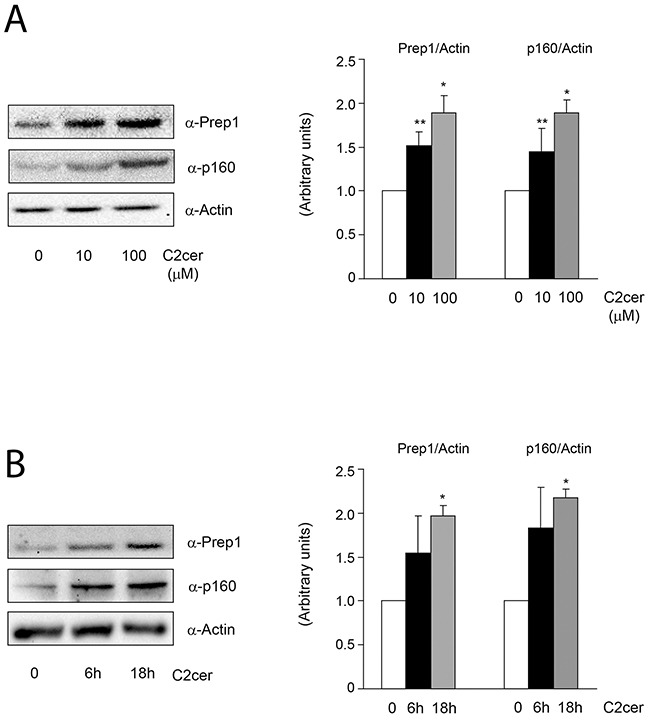
Effect of ceramides dose-response and time-course on Prep1 and p160 protein levels L6 skeletal muscle cells were treated with different concentration of C2cer (10μM and 100μM for 18h) **(A)** and for different time (10μM for 6h and 18h) **(B)**. Protein lysates were analyzed by Western blot using antibodies for Prep1, p160 and for the beta-actin, as a loading control. Blots were detected by ECL and autoradiography. The autoradiographs are representative of four independent experiments. Asterisks denote statistically significant differences (*p<0.05; **p<0.01).

**Figure 2:**
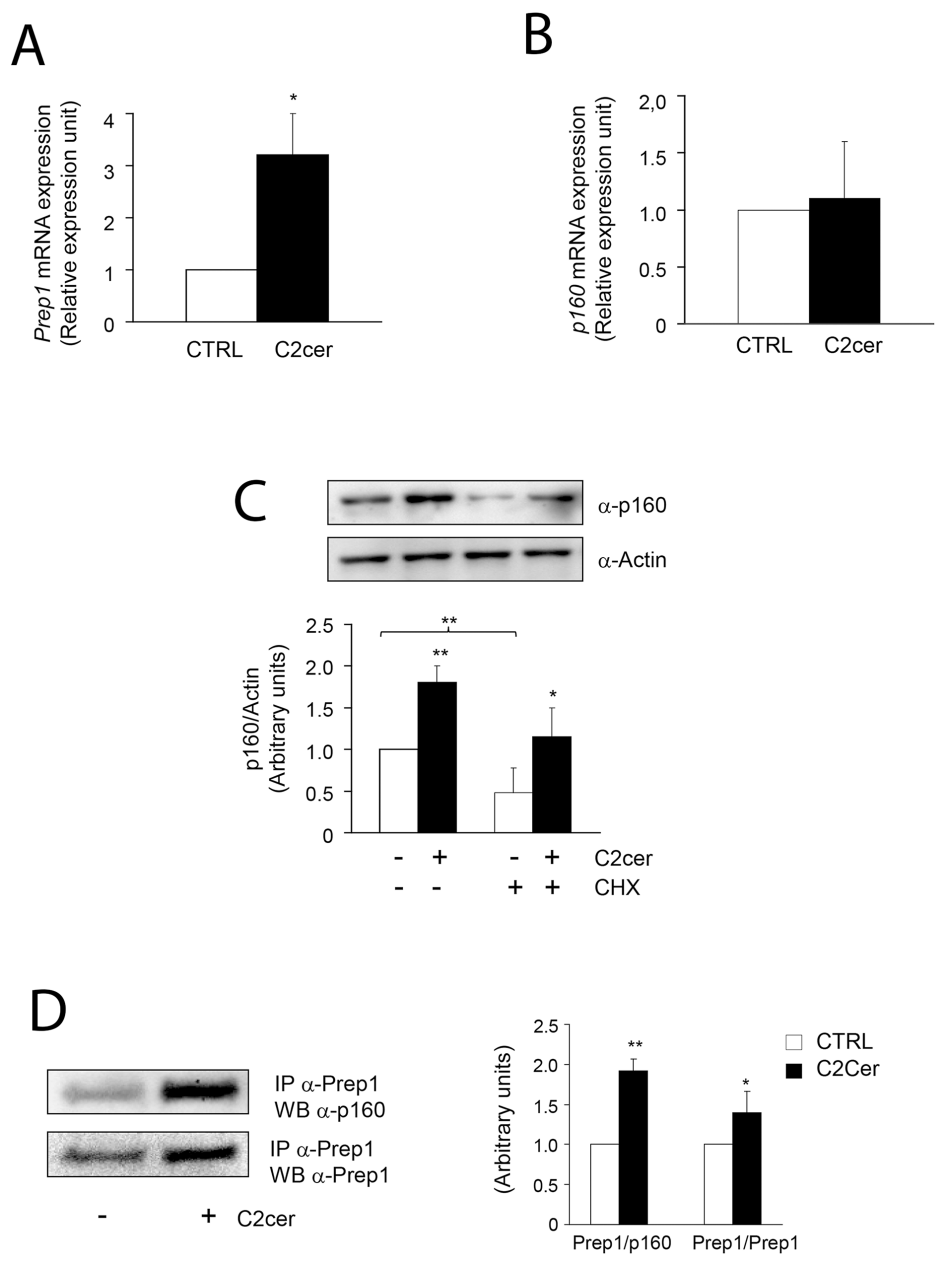
Effect of ceramides on Prep1 and p160 mRNA expression and of Prep1-p160 coprecipitation L6 cells were treated with C2cer (10μM) for 18h and Prep1 **(A)** or p160 **(B)** mRNA levels were analyzed by real-time RT-PCR analysis. Data were normalized by the amount of GAPDH mRNA, used as internal control. Bars represent the mean ± SD of three independent experiments, each performed in triplicate. **(C)** L6 cells were treated with cycloheximide (CHX) at 40μg/mL concentration for 18h and the lysates were analyzed by Western blot with anti-p160 and beta-actin. The autoradiograph shown is representative of three different experiments and subjected to densitometric analysis. **(D)** Protein lysates from L6 cells treated with C2cer as indicated, were immunoprecipitated with Prep1 antibody and immunoblotted with p160 or Prep1 antibodies. The autoradiograph shown is representative of three different experiments and subjected to densitometric analysis. Asterisks denote statistically significant differences (*p<0.05; **p<0.01).

We first measured the levels of Prep1 and p160 in L6 cells incubated with different amounts of C2cer. As shown in Figure 1A, C2cer increased Prep1 and p160 by 50 and 90% at 10 and 100μM, respectively. However, we noted that the highest concentration was slightly toxic for the cells (data not shown), thus, 10μM C2cer was used for all further experiments. Time-dependent effects of ceramides treatment on Prep1 and p160 showed that protein levels raised up at 18 hours exposure (Figure 1B). Similar result was obtained after incubating L6 cells with palmitate (PAL) as described in Supplementary Figure 1. C2cer increased also the mRNA levels of Prep1 by 3.2-fold (Figure 2A), but, at variance, p160 mRNA levels were not affected by C2cer leading to the hypothesis that p160 protein increase was due to a Prep1-mediated p160 stabilization as reported by Oriente et al [18] (Figure 2B). To confirm this hypothesis, L6 cells were incubated with the protein synthesis inhibitor cycloheximide (CHX) (40μg/ml). We found that this treatment reduced p160 cellular levels by 50%. However, p160 protein levels were induced by C2cer even in the presence of CHX (Figure 2C). Interestingly, C2cer not only increased total Prep1 and p160 protein levels, but also induced co-precipitation between the two proteins (Figure 2D).

### Prep1(54-72) prevents ceramide-induced Prep1/p160 complex formation

**Figure 3:**
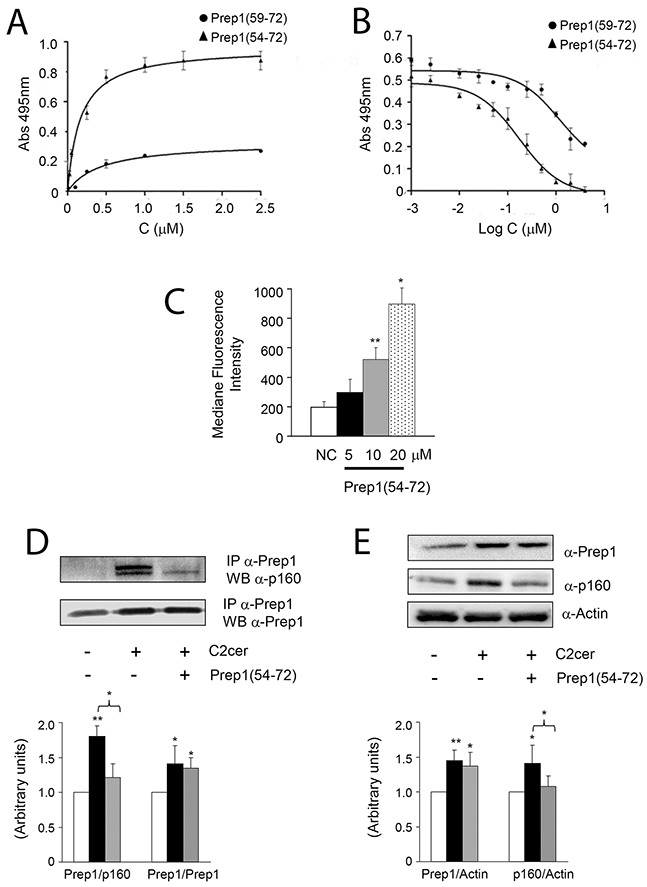
Design and *in vitro* effects of Prep1(54-72) peptide on Prep1/p160 association **(A)** Binding curves of Prep1(59-72) and Prep1(54-72) peptides to p160_20-160_ by ELISA-like assays. Biotinylated peptides at several concentrations were added in triplicate to a 96-well plate coated with p160_20-160_ (0.1μM). Experiments were performed at least twice and the average results fitted using GraphPad Prism. Fitting of data with a one-site binding model provided a KD value of 0.18μM ± 0.04 and 1.25μM ± 0.08 for Prep1(54-72) and Prep1(59-72) respectively. **(B)** Competition binding experiments. His6-tagged-p160_20-160_ at 0.25μM was incubated with Prep1(59-72) and Prep1(54-72) at concentrations ranging between 0.001÷10μM and added to a 96-well plate coated with Prep1_45-155_ (0.10μM). Results are the average of three independent experiments. **(C)** L6 cells were incubated with increasing concentrations of the fluorescein-conjugated peptide (FITC-Prep1(54-72)) and peptide uptake was detected by FACS analysis of fluorescein-labeled cells. Bars represent the mean ± SD of four independent experiments. **(D)** L6 muscle cells incubated with Prep1(54-72) were stimulated with C2cer (10μM) for 18h. Protein lysates were immunoprecipitated with Prep1 antibodies and analyzed by Western blot using antibodies for p160 or for Prep1. **(E)** An aliquot of the lysate was used to measure protein expression of Prep1 and p160 and the beta-actin used as loading control. The blots were detected by ECL and autoradiography. The autoradiograph is representative of three independent experiments. Asterisks denote statistically significant differences (*p<0.05; **p<0.01).

To address the significance of the association between Prep1 and p160 mediated by C2cer, we generated a synthetic inhibitor of the Prep1/p160 interaction. Experimental evidences have suggested that region 63-70 (^63^LFPLLALL^70^) of the HR1 domain of Prep1 mediates the interaction with p160 [21]. The synthetic peptide covering such stretch of residues was therefore chosen to generate a potential inhibitor. Secondary structure prediction studies suggested that this protein region has strong propensity to form helical structures [22]. Thus, we designed and chemically synthesized a peptide spanning residues 59-72, hereafter Prep1(59-72), which comprises the predicted α-helix and a short loop at the N-terminus (Supplementary Figure 2A, upper panel). We also designed and prepared an additional peptide with a slightly longer N-terminal end, named Prep1(54-72) (Supplementary Figure 2A, lower panel), to increase the probability of obtaining a molecule with helical features. Peptide sequences and their experimental and theoretical MWs are reported in Table 1. CD spectroscopy studies showed that the two peptides did not adopt ordered structures in aqueous buffers (Supplementary Figure 2B), but they converted to alpha-helical conformations after the addition of only 20% TFE (Supplementary Figure 2C and 2D). In order to test the affinity of Prep1 synthetic peptides to p160 protein, ELISA-like experiments were developed where N-terminally biotinylated peptide variants were added to p160_20-160_ – coated 96-well plates [22]. As shown in Figure 3A either variants bound p160_20-160_ domain in a dose-dependent and saturable manner. However, by fitting the binding curves with a non-linear regression algorithm, very different affinity values were extrapolated. Indeed, Prep1(54-72) and Prep1(59-72) bound p160_20-160_ with KDs of 0.18μM and 1.25μM, respectively. Moreover, despite the same amount of p160_20-160_ was coated on the plate surface, a considerably lower amount of Prep1(59-72) was bound compared to Prep1(54-72), suggesting an overall reduced affinity displayed by the shorter peptide. To further assess the binding specificity, competition experiments were carried out employing Prep1(54-72) and Prep1(59-72) as competitor of the p160_20-160_/Prep1_45-155_ binding (Figure 3B). Prep1(54-72) inhibited the binding between p160_20-160_ and Prep1_45-155_ in a dose-dependent fashion and data fitting provided an IC_50_ of 0.20μM, consistent with the K_D_ determined by direct binding. Prep1(59-72) also suppressed the binding between the two proteins at the highest concentration of 10μM, however the signal was not totally abolished and fitting of experimental data did not converge to a meaningful value (Figure 3B), in agreement with the lower affinity of this peptide for the p160_20-160_ domain. These results show that Prep1(59-72) displays a reduced affinity for p160_20-160_ compared to Prep1(54-72) variant, and suggest an important role of the N-terminal residues in the interaction or in the stabilization of a more favorable p160-binding conformation.

**Table 1:** Single letter sequences and molecular weights (calculated and experimental) of peptides Prep1(54-72) and Prep1(59-72) used in this study

Peptide	Sequence	MWcalc(amu)	MWexp(amu)
Prep1(54-72)	Ac-KQAIYRHPLFPLLALLFEK-NH2	2339.8	2338.6
Prep1(59-72)	Ac-RHPLFPLLALLFEK-NH2	1736.10	1735.6

Thus, Prep1(54-72) was chosen for further investigations in L6 cells. FACS analyses with a FITC-conjugated analogue showed that the peptide could be vehiculated in a dose-dependent manner into cells in the presence of liposomes and that transduction was efficient yet at 10μM without inducing any apparent cell toxicity (Figure 3C and data not shown). We then tested whether the peptide blocked the interaction between the two proteins in L6 cells. Prep1-p160 co-precipitation stimulated by ceramides was almost completely reverted by Prep1(54-72) (Figure 3D), suggesting that the peptide bound the full-length protein also in cells. In addition, Prep1(54-72) did not modify the ceramide-induced Prep1 levels, while decreased those of p160 by 25% (Figure 3E).

### Prep1-p160 interaction is important for ceramide action

**Figure 4:**
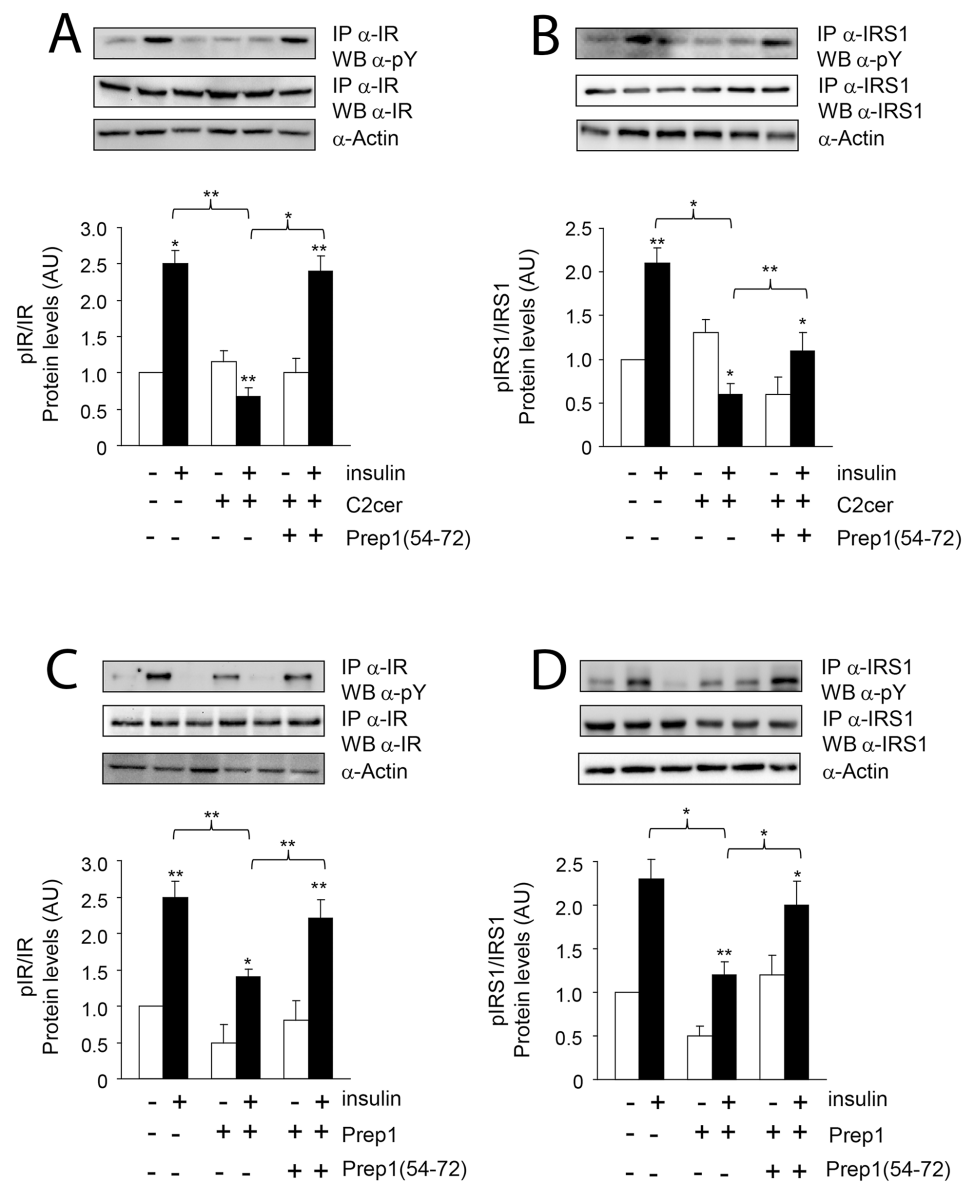
Prep1(54-72) peptide restores the insulin signaling inhibited by C2cer and Prep1 L6 cells were stimulated with insulin (100nM) and incubated with Prep1(54-72) in presence of C2cer at 10μM for 18h **(A-B)** or stable transfection of Prep1 **(C-D)**. Protein lysates were immunoprecipitated with specific IR or IRS1 antibodies and analyzed by Western blot using antibodies for phosphotyrosines (pY) and beta-actin as loading control. Blots were detected by ECL and autoradiography. The autoradiographs are representative of three independent experiments. Asterisks denote statistically significant differences (*p<0.05; **p<0.01).

**Figure 5:**
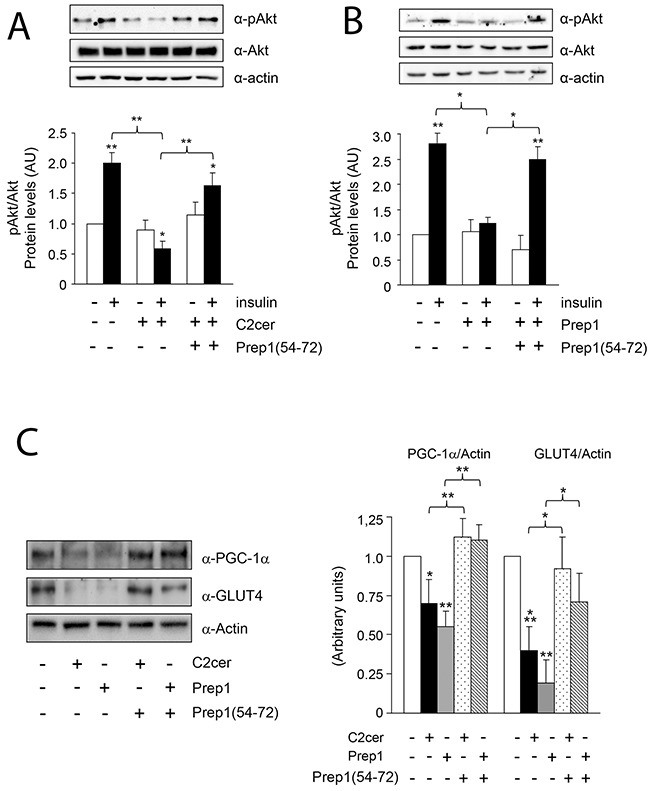
Effect of Prep1(54-72) peptide on Akt phosphorylation and PGC-1α and GLUT4 protein levels **(A-B)** L6 control and Prep1 overexpressing cells were stimulated with Insulin (100nM) and/or C2cer (10μM) and incubated with Prep1(54-72). Protein lysates were analyzed by Western blot using antibodies for pAkt, Akt and for the beta-actin, as a loading control. **(C)** PGC-1α and GLUT4 protein levels were measured by using specific antibodies for these proteins and beta-actin antibody was used as loading control. Blots were detected by ECL and autoradiography. The autoradiographs are representative of four independent experiments. Asterisks denote statistically significant differences (*p<0.05; **p<0.01; ***p<0.001).

**Figure 6:**
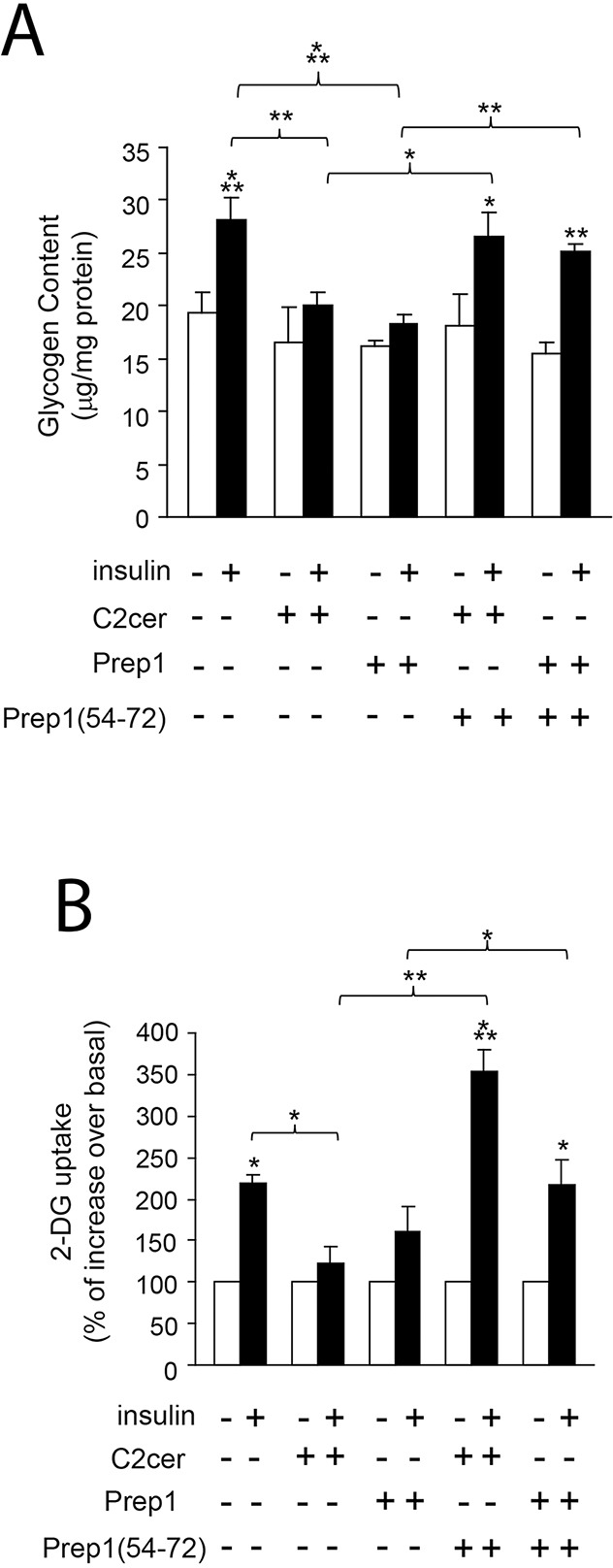
Effect of Prep1(54-72) peptide on glycogen content and 2-DG uptake L6 control and Prep1 overexpressing cells were stimulated with Insulin (100nM) and/or C2cer (10μM) and incubated with Prep1(54-72). **(A)** Glycogen content and **(B)** 2-DG uptake were measured as described in methods. Bars represent the mean ± SD of three independent experiments. Asterisks denote statistically significant differences (*p<0.05; **p<0.01; ***p<0.001).

Previous data suggested Prep1 as a potential mediator of metabolic response in several tissues [18–20] and ceramides as promoters of insulin resistance in skeletal muscle [4–6]. On these bases, we investigated the effects of Prep1 induction by ceramides on insulin signaling and those produced by disrupting its interaction with p160. In L6 cells, insulin stimulated IR and IRS1 tyrosine phosphorylation by 1.5- and 1.1-fold and this effect was blocked by C2cer. Interestingly, treatment with Prep1(54-72) restored insulin-stimulated IR and IRS1 phosphorylation (Figure 4A and 4B). In parallel, L6 cells stably transfected with the *Prep1* cDNA (Prep1L6) showed a 50 and 45% reduction of basal and insulin-stimulated IR and IRS1 tyrosine phosphorylation, respectively. However, when Prep1L6 cells were incubated with Prep1(54-72), IR and IRS1 were stimulated similarly to the control cells (Figure 4C and 4D). According to these data, insulin stimulated-Akt phosphorylation was not significantly induced by insulin in presence of either ceramides or Prep1 transfection. In contrast, treatment with Prep1(54-72) rescued insulin effect (Figure 5A-5B). Similarly, PGC-1α and GLUT4 protein expression, previously reported to be regulated by Prep1 [18], decreased by approximately 35 and by 60% after incubation with C2cer and Prep1, respectively, and administration of Prep1(54-72) significantly reverted this effect (Figure 5C). To further address the functional consequences of blocking Prep1-p160 interaction on insulin signaling, we measured glycogen content and 2-DG uptake. Insulin stimulated glycogen synthesis and glucose uptake by 1.3- and 2.1-fold, respectively and these effects were negatively modulated by C2cer and Prep1. Incubation of L6 cells with Prep1(54-72) recovered the insulin response (Figure 6A-6B).

## DISCUSSION

Hyperlipidemia is a pivotal factor contributing to insulin resistance. However, the type and the mechanisms by which excess lipid downregulates insulin action are unclear. For example, skeletal muscle of trained endurance athletes is markedly insulin sensitive, despite having an elevated free fatty acids (FFAs) content [23]. Among the FFAs, palmitate has been considered extremely dangerous as it contributes to insulin resistance by inducing the synthesis of several lipid-derived intermediates like ceramides [2–7]. This class of lipids is one of the main mediators of palmitate-induced insulin signaling impairment. Indeed, incubation of several types of skeletal muscle cells with palmitate significantly increases ceramide levels leading to inhibition of insulin pathway. These data are further supported by other experiments showing that inhibitors of serine palmitoyl transferase, a key enzyme that commits palmitate to the *de novo* synthesis of ceramide, revert the palmitate-induced inhibition of glucose uptake in skeletal muscle cells [24–26]. In parallel, both short and long period ceramide exposition inhibit insulin-stimulated glucose uptake, GLUT4 translocation and glycogen synthesis by suppressing the tyrosine phosphorylation of IRS1 or the Akt activation [3, 5, 8]. All these observations indicate ceramides as particularly harmful molecules in the development of insulin resistance.

We have previously demonstrated that the transcription factor Prep1 is a physiologic regulator of insulin-mediated glucose and lipid metabolism in skeletal muscle and in liver [18–20], and its expression is enhanced by high glucose levels [27].

In the current work, we have examined the role of Prep1 on ceramide-mediated skeletal muscle insulin resistance.

L6 cells treated with ceramides feature a dose-response increase of Prep1 and p160 protein levels reaching a statistical significance already at 10μM. In parallel, time-course experiments show that Prep1 and p160 expression increases after 6 hours exposure to C2cer, but the maximum and significant effect is achieved after 18 hours. Analogous effects are observed in presence of palmitate, however, as described above, we have focused our particular attention on the role of ceramide as these sphingolipids mediate the responses of palmitate. Interestingly, Prep1 expression is induced by C2cer also at mRNA level. Our preliminary data indicate that a specific NF-κB pharmacologic inhibitor (JSH-23) reverts the effect of C2cer on P*rep1* mRNA expression and this result is also consistent with reports by Demarchi et al [28] and by Ciccarelli et al [27] indicating NF-κB as a downstream mediator of C2cer and an inducer of *Prep1* gene expression. However, this point needs further clarifications and is actually under investigation in our laboratory. Unlike Prep1, *p160* gene expression does not increase in presence of ceramides. L6 myotubes treated with the protein synthesis inhibitor cycloheximide feature reduced p160 cellular levels, but this effect is attenuated in presence of the C2cer, suggesting that the mechanism responsible for the ceramide-p160 increase may be posttranslational. As previous data have shown that Prep1 interaction stabilizes p160 and induces p160 escape from proteasomal degradation [21], we have demonstrated that C2cer-mediated p160 protein expression may involve Prep1-p160 interaction. To further support this concept, we have developed a peptide that disrupts the interaction and restores the mechanisms associated with complex formation. The peptide has been designed on the basis of previous studies [21] showing that the minimum interacting region of Prep1 on p160 includes a polyleucine stretch encompassing residues 63-70 and that this region adopts alpha-helical conformations [22]. We end up with an optimized synthetic peptide that spans region 54-72 of Prep1 that *in vitro* is able to bind to p160 and to displace its binding with Prep1 in biochemical assays as well as in cells overexpressing Prep1. As expected, Prep1(54-72) has a strong propensity to adopt a pretty helical conformation in solution. Furthermore, incubation of the L6 cells with this peptide largely reduces the C2cer-induced Prep1-p160 coprecipitation. Interestingly, in presence of the peptide also the levels of p160 decrease, furtherly supporting the hypothesis that ceramides regulate p160 amounts through the binding with Prep1. These data suggest that the mechanism by which Prep1(54-72) reduces the Prep1-p160 coprecipitation involves a direct protein-protein binding displacement. However, we cannot rule out a direct action on reducing p160 protein levels.

To study a possible role of the peptide on insulin signaling, we incubated L6 cells, treated with ceramides or overexpressing Prep1 and stimulated with insulin, with Prep1 (54-72). Long-term exposure of L6 cells with C2cer or Prep1 overexpression strongly reduced insulin-mediated IR, IRS1 and Akt phosphorylation as well as PGC-1α and GLUT4 protein expression, leading to the inhibition of glycogen synthesis and glucose uptake. Interestingly, peptide treatment restored insulin response, suggesting that the Prep1-p160 complex might be a privileged target for improving insulin sensitivity in individuals with high FFAs content and metabolic alterations.

**Figure 7:**
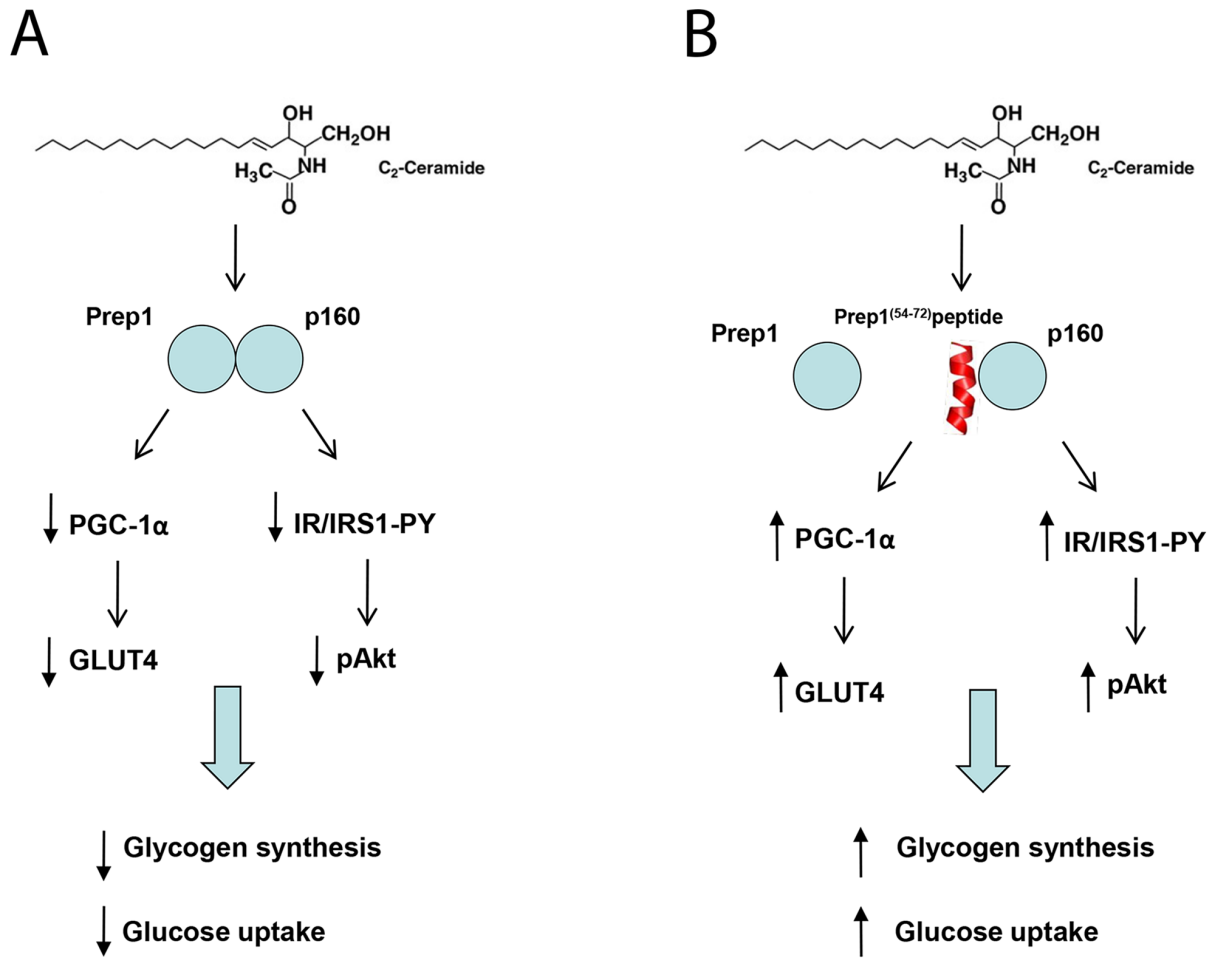
Schematic representation of Prep1(54-72) peptide action on insulin signaling impaired by ceramides **(A)** Ceramides increase both Prep1 and p160 expression and induce the coprecipitation between the two proteins. Prep1/p160 complex impairs insulin signaling and reduces PGC-1α and GLUT4 expression, leading to a decrease of glycogen synthesis and 2DG-uptake. **(B)** Incubation of the L6 cells with Prep1(54-72) (in red) mimicking the region of Prep1 which interacts with p160, displaces the protein-protein binding and improves the ceramide-mediated insulin signaling impairment.

In conclusion, our data indicate that, in L6 cells, C2cer promote Prep1-p160 association and impairs metabolic effects through two distinct mechanisms, one involving the downregulation of PGC-1α and GLUT4, the other, involving an impairment of IR-IRS1 tyrosine phosphorylation. Remarkably, the Prep1(54-72)-mediated Prep1-p160 complex disruption rescues both pathways and restores insulin signalling (Figure 7A-7B). Data thus support the view that individuals overexpressing one of the two proteins may undergo poor insulin sensitivity and that selective and efficient inhibition of the complex can be highly beneficial in terms of a more effective glucose utilization and consumption in muscle tissues. Interestingly, ceramides are known to induce the activation of the c-Jun N-terminal kinase (JNK), an essential regulator of physiological and pathological processes [29, 30]. JNK mediates the effect of stress on insulin resistance through inhibitory phosphorylation of insulin receptor substrates [31]. Thus, it is not surprising that suppression of the JNK pathway improves insulin resistance and glucose tolerance. However, the pharmacological inhibition of JNK might not represent a successful therapeutic strategy, since it interferes with both deleterious and beneficial functions of JNK [32–34]. In contrast, delivering molecules able to selectively inhibit the Prep1-p160 interaction may offer relevant benefits in terms of reduced side effects and specific enhancement of insulin sensitivity.

Because of the expected poor stability and bioavailability profiles featuring all-L linear peptides, Prep1(54-72), used here as a model compound for complex inhibition, is markedly not ideal for such application *in vivo* or for further developments as a drug. However, given the relatively high potency *in vitro* (IC_50_ ≈ 200nM) and the specificity for p160, it could serve as a valuable scaffold for designing new, more effective and potent complex inhibitors. These future studies might be also relevant to evaluate the role of Prep1 inhibition in humans. Indeed, although we still do not have data from human skeletal muscle biopsies, our preliminary experiments indicate that peripheral blood leucocytes from first-degree relatives of type 2 diabetics, with a very high risk of type 2 diabetes and known to be insulin-resistant [35], overexpress *PREP1* gene by 3.5-fold compared to control individuals with no family history of diabetes.

## MATERIALS AND METHODS

### Materials

Media, sera, antibiotics for cell culture and the Lipofectamine reagent were all from Invitrogen (Grand Island, NY, USA). Ceramide D-erythro-Sphingosine, N-Acetyl- (C_2_ Ceramide), pY and IRS1 antibodies were from Merck Millipore (Darmstadt, Germany). Cycloheximide (CHX), palmitate and Biotin Reagents were obtained from Sigma-Aldrich (Darmstadt, Germany). Prep1, PGC-1α, pAkt, Akt and actin antibodies were from Santa Cruz Biotechnology (Santa Cruz, CA, USA). IR antibody was from Cell Signaling Technology (Beverly, MA, USA). p160 antibody was purchased from Zymed Laboratories (San Francisco, CA, USA). Glucose Transporter 4 (GLUT4) was from Abcam In. (Cambridge, MA). Protein electrophoresis and real-time PCR reagents were from Bio-Rad (Richmond, VA, USA). Western blotting and ECL reagents were purchased from Amersham Biosciences (Arlington Heights, IL, USA). Reagents for peptide synthesis including Fmoc-protected amino acids and resins, activation and deprotection reagents, were purchased from Calbiochem-Novabiochem (Laufelfingen, Switzerland) and Sigma-Aldrich (Darmstadt, Germany). Solvents for peptide synthesis and HPLC analyses were purchased from Delchimica (Naples, Italy); C18 Biobasic columns for peptide analysis and the LC-MS system were from ThermoFisher (Milan, Italy).

### Cell culture procedures and prep1 stable transfection

L6 rat skeletal muscle cells (ATCC, Manassas, VA, USA) were cultured in DMEM supplemented with 10% FBS and 1% penicillin/streptomycin solution at 37°C in a humidified 95% air and 5% CO_2_ atmosphere (all vol./vol.). Prep1 stable transfection in L6 cells has been described elsewhere [27].

### Chemical synthesis and purification of peptides

Solid phase peptide synthesis was performed on a fully automated multichannel peptide synthesizer Syro I (Multisyntech, Germany) following the Fmoc methodology [36]. Preparative RP-HPLC purification was carried out on a Waters Quaternary Gradient Module, equipped with a Waters UV/Vis detector and with a XBridgeTM Prep BEH 130 column (19×50mm; 185μm). MS analyses were carried out on a Bruker HCT ETD II ultra PTM Discovery System equipped with an ESI source maintained at 5 kV and 300°C. Narrow bore 50×2 mm, C18 BioBasic LC-MS columns were used for these analyses. Prep1 peptides were biotin labelled using Biotin Reagents, following the producer protocol; LC-MS analysis was used to confirm peptide derivatization. Prep1_45-155_ and His6-tagged p160_20-160_ (His6-p160_20-160_) were prepared as described in [22].

### ELISA-like assays

ELISA experiments were performed using an integrated platform for High-Throughput Screening (Hamilton Robotics, Bonaduz, CH) comprising a fully equipped Starlet 8 channel liquid handler, a robotic arm, a washer and a multi-wavelength plate reader. ELISA assays were performed as previously reported, introducing slight modifications [22]. In particular, several concentrations of biotinylated peptides at several concentrations were added to a plate coated with p160_20-160_ (0.1μM) and binding was detected with streptavidin-HRP. The competitive binding experiments were carried out by coating with PREP1_45-155_ (0.1μM) and pre-incubating the His6-tagged-p160_20-160_ at 0.25μM with peptides in dose-response experiments. The binding was detected by anti-His HRP-conjugated antibody. Data were fitted with GraphPad Prism (GraphPad Software Inc, San Diego, California). Binding curves were best fitted with one-site binding models.

### Western blot analysis and Immunoprecipitation procedures

Total cell lysates were obtained and separated by SDS-PAGE. Briefly, cells were solubilized with lysis buffer containing 50mM HEPES, 150mM NaCl, 10mM EDTA, 10mM Na_4_P_2_O_7_, 2mM sodium orthovanadate, 50mM NaF, 1mM phenylmethylsulfonyl fluoride, 10μg/ml aprotinin, 10μg/ml leupeptin, pH 7.4, and 1% (v/v) Triton X-100. Lysates were clarified by centrifugation at 12,000g for 20 minutes at 4°C. The protein concentrations in the cell lysates were measured using a Bio-Rad DC (detergent compatible) assay. Immunoprecipitation and Western blot analysis have been performed as previously described [37].

### Real-Time RT-PCR analysis

Total cellular RNA was isolated from L6 cells by using the RNeasy kit (QIAGEN Sciences, Germany), according to manufacturer instructions. 1μg of cell RNA was reverse-transcribed using Superscript III Reverse Transcriptase (Life Technologies Carlsbad, CA, USA). PCR reactions were analyzed using IQTM SYBR Green Supermix (Bio-Rad, Hercules, CA). Reactions were performed using Platinum SYBR Green qPCR Super-UDG using an iCycler IQ multicolor Real Time PCR Detection System (Biorad, CA). All reactions were performed in triplicate and GAPDH was used as an internal standard. Primer sequences used are described in electronic Supplementary Material (ESM) Supplementary Table 1.

### Fluorescence-activated cell sorting (FACS) analysis

L6 cells were incubated with negative control (NC) and Prep1(54-72) peptides conjugated with fluorescein isothiocianate (FITC) at different concentrations and harvested 48h later. Then, pellet was fixed with 3% formaldehyde for 15 min at 37°C, washed three times and resuspended in PBS for 20 min and the emission in FL-1 channel was analyzed. The samples were acquired by a BD LSRFortessa (BD Bioscience, San Jose, CA) and analyzed using BD FACS Diva Software.

### Glycogen content and 2-DG uptake analyses

Glycogen was isolated from L6 cells homogenized in 0.1% SDS, saturated with Na_2_SO_4_ for 30 minutes at 37°C, followed by ethanol (EtOH) precipitation. Glycogen content was determined as previously described [19]. 2-DG uptake by the L6 cells was measured as previously reported [18].

### Statistical procedures

Data were analyzed with the GraphPad Prism 6.0 by unpaired two-tailed *t-*test and one way Anova followed by Sidak's multiple comparison tests. *p* values of less than 0.05 were considered statistically significant [38].

## SUPPLEMENTARY MATERIALS FIGURES AND TABLE



## Abbreviations

2-DG uptake: 2-Deoxyglucose uptake
C2cer: C2 ceramides
CHX: cycloheximide
GLUT4: glucose transporter 4
IR: insulin receptor
IRS1: insulin receptor substrate 1
p160: p160 myb binding protein
PAL: palmitate
PGC-1α: Peroxisome proliferator-activated receptor gamma coactivator-1alpha
Prep1: Pbx regulating protein 1.
