# Supplementary material for: A peptide antagonist of Prep1-p160 interaction improves ceramide-induced insulin resistance in skeletal muscle cells
Source: Oncotarget. 2017 May 30;8(42):71845–58. doi: 10.18632/oncotarget.18286 (PMC5641094; doi:10.18632/oncotarget.18286)
Supplement: Supplementary file 1 [file oncotarget-08-71845-s001.pdf]

A peptide antagonist of Prep1-p160 interaction improves ceramide-induced insulin resistance in skeletal muscle cells

SUPPLEMENTARY MATERIALS

REFERENCES

1. Lorenzo V, Mascanzoni F, Vitagliano L, Ruvo M and Doti N. The Interacting Domains of PREP1 and p160 are Endowed with a Remarkable Structural Stability. Mol Biotechnol. 2016; 58:328-339.

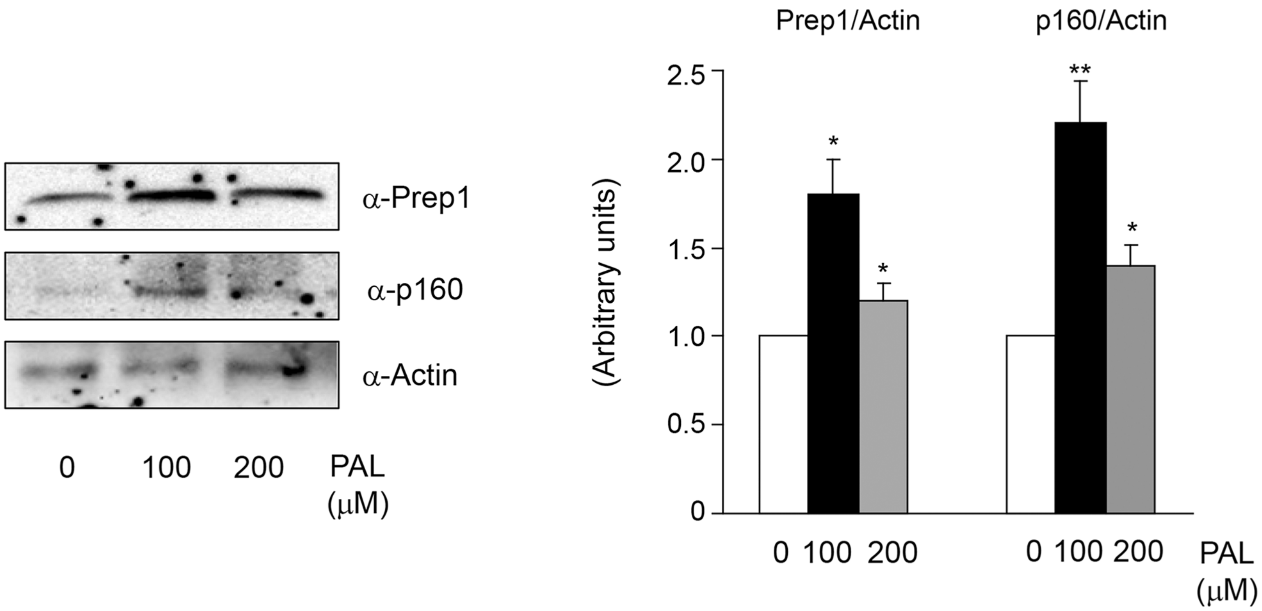

Supplementary Figure 1: Effect of palmitate on Prep1 and p160 protein levels. L6 skeletal muscle cells were treated with different concentration of palmitate (PAL) for 18h. Protein lysates were analyzed by Western blot using antibodies for Prep1, p160 and for the beta-actin, as a loading control. Blots were detected by ECL and autoradiography. The autoradiographs are representative of four independent experiments. Asterisks denote statistically significant differences (*p<0.05; **p<0.01).

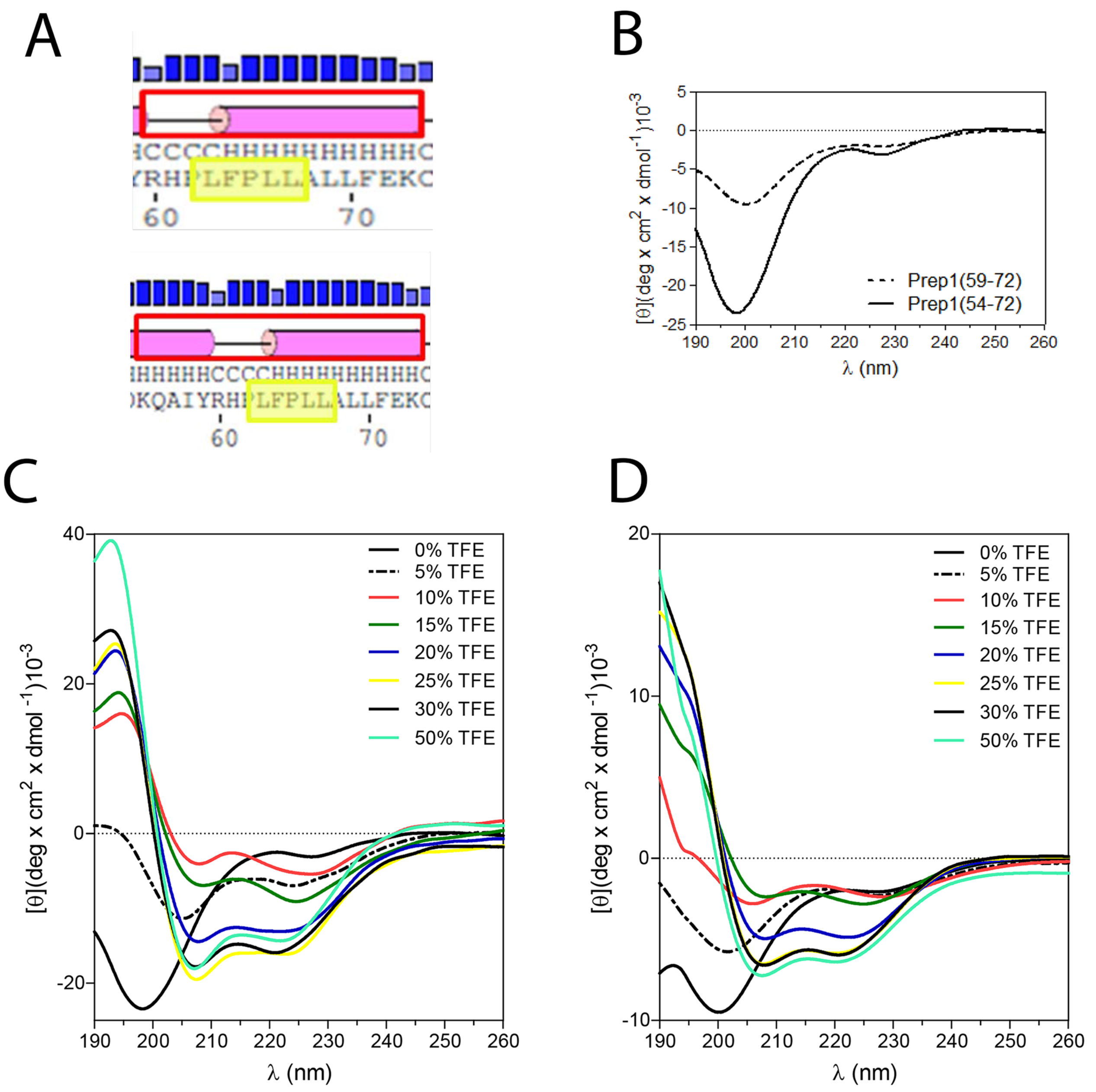

Supplementary Figure 2: Structural prediction and characterization of Prep1 peptides. **(A)** Secondary structure prediction of Prep1 amino acidic region spanning residues 59-72 (upper panel) and 54-72 (lower panel), using the PSIPRED server (http://bioinf.cs.ucl.ac.uk/psipred/), as reported in literature [1]. **(B)** Far-UV CD spectra of Prep1(59-72) (dashed line) and Prep1(54-72) (continuous line) peptides recorded in 5mM phosphate buffer, pH 7.4, at 20 °C. **(C, D)** TFE-titration curves recorded in 5mM phosphate buffer, pH 7.4, at 20 °C of Prep1(54-72) and Prep1(59-72), respectively.

Supplementary Table 1: Sequences of primers used in this study

Prep1	F: 5′-GGAGTGCCAACCATGTTAAGAAGAAGTCCC-3′<br>R: 5′-GACACCGTGTGCTTCTCGCTCAAG-3′
p160	F: 5′-AGACAAGCAATGTACCGACTACAG-3′<br>R: 5′-GGCTCTGGTGGACATCCTCTC-3′
GAPDH	F: 5’-CTTGACTGTGCCGTTGAACT-3’<br>R: 5’-ATGGTGAAGGTCGGTGTGAA-3’
